# An *in vitro* erythrocyte preference assay reveals that *Plasmodium falciparum* parasites prefer Type O over Type A erythrocytes

**DOI:** 10.1038/s41598-018-26559-2

**Published:** 2018-05-25

**Authors:** Michel Theron, Nadia Cross, Paula Cawkill, Leyla Y. Bustamante, Julian C. Rayner

**Affiliations:** 1 Malaria Programme, Wellcome Sanger Institute, Wellcome Genome Campus, Hinxton, Cambridge, CB10 1SA United Kingdom; 2 Present Address: Ferrier Research Institute, Chemical Biology Laboratory, Kelburn Parade, Victoria University of Wellington, PO Box 600, Wellington, 6140 New Zealand; 3 Present Address: F. Hoffman-La Roche Ltd, Hochstrasse 16, 4053, Basel, Switzerland

## Abstract

Malaria has been one of the strongest selective forces on the human genome. The increased frequency of haemoglobinopathies, as well as numerous other blood groups, in malaria endemic regions is commonly attributed to a protective effect of these alleles against malaria. In the majority of these cases however there have been no systematic functional studies to test protective mechanisms, in large part because most host-parasite interaction assays are not quantitative or scalable. We describe the development of an erythrocyte preference assay which uses differential labelling with fluorescent dyes to distinguish invasion into four different erythrocyte populations which are all co-incubated with a single *Plasmodium falciparum* parasite culture. Testing this assay on erythrocytes across the ABO blood system from forty independent donors reveals for the first time that *P. falciparum* parasites preferentially invade group O over Group A erythrocytes. This runs counter to the known protective effect of group O against severe malaria, but emphasises the complexities of host-pathogen interactions, and the need for highly quantitative and scalable assays to systematically explore them.

## Introduction

The impact of malaria parasites on human erythrocyte biology has been clear ever since the publication of the Haldane hypothesis in 1949, which proposed that β-thalassemia is prevalent in some parts of the world because the negative impact of severe anemia in homozygotes is counter balanced by protection from severe malaria in heterozygotes^1^. In the following decades numerous erythrocyte variants, particularly haemoglobinopathies, have been associated with protection against severe malaria, with some variants such as HbAS providing up to 95% protection^2^. Large scale genome-wide association studies are now adding many more malaria protective alleles^3^, including variants near the major erythrocyte surface calcium ATPase^4^, and structural variation at a locus that encodes the major erythrocyte surface sialoglycoproteins, Glycophorin A and B^5^.

Surprisingly, even for variants that have a well-documented and defined protective effect against malaria such as sickle-cell trait, the cellular and molecular mechanisms of that protection are often either unknown or disputed^6^. This is due in part to technical challenges associated with functional assays - testing the ability of *P. falciparum* strains to invade or replicate inside human erythrocytes from different genetic backgrounds requires highly quantitative assays and preferably ones in which erythrocytes from multiple backgrounds can be compared directly and simultaneously. Few such assays exist. Sickle-cell trait is a useful case in point, where detailed *in vitro* studies have variously assigned the protective mechanism to a reduction in growth in AS erythrocytes under hypoxic conditions due to various inhibitory effects of HbS polymers^7^, decreased adherence of AS infected erythrocytes to the endothelium and hence increased clearance by the spleen^8^, and most recently differential expression of parasite growth inhibitory microRNAs in AA and AS erythrocytes^9^. Similarly, another variant of beta haemoglobin, HbC, has been proposed to protect against malaria by inhibiting rupture of CC infected erythrocytes^10^, by causing spontaneous loss of parasite subcellular compartmentalization, or by a decrease in cytoadherence^11^. These sometimes conflicting findings are all based on detailed mechanistic studies, but because of throughput limitations, these studies frequently rely on only a small number of erythrocyte samples, where it is difficult to disentangle the potential confounding impact of genetic background. There is a clear need for more robust, and scalable, assays that allow direct comparison of parasite phenotypes in erythrocytes from multiple different sources.

We previously developed a method to measure *P. falciparum* invasion using fluorescently labelled erythrocytes^12^. Erythrocyte labelling can be used to distinguish invasion into labelled or “target” erythrocytes from erythrocytes that were present in the starting parasite culture or “donor” erythrocytes, while parasitemia in both donor and target populations can be quantitated using a labelled DNA dye that emits at a different fluorescent wavelength to the erythrocyte dye. The approach the advantage of being scalable, requires no manipulation of the parasite culture prior to setting up the assay, and is highly accurate even at low parasitemia. It has been subsequently used in *ex vivo* studies of erythrocyte invasion^13,14^, has been adapted for other *Plasmodium* species^15^, and further developed to quantify invasion into two different erythrocyte populations^16^. In this study we sought to expand the approach to more complex systems such as human blood groups, where erythrocyte invasion rates would need to be compared across multiple human genetic backgrounds. We tested several different dyes and developed an assay where *P. falciparum* parasites are co-incubated with fluorescently labelled erythrocytes from multiple donors within a single well, and fluorescent DNA staining is used to quantitate parasitemia into each population separately. The resulting assay, which we refer to as an erythrocyte preference assay, is able to concurrently measure *P. falciparum* invasion rates into four different erythrocyte populations, and we used it to systematically test invasion preferences across the ABO blood group.

## Results

### Development of a flow cytometry based erythrocyte preference assay

**Figure 1:**
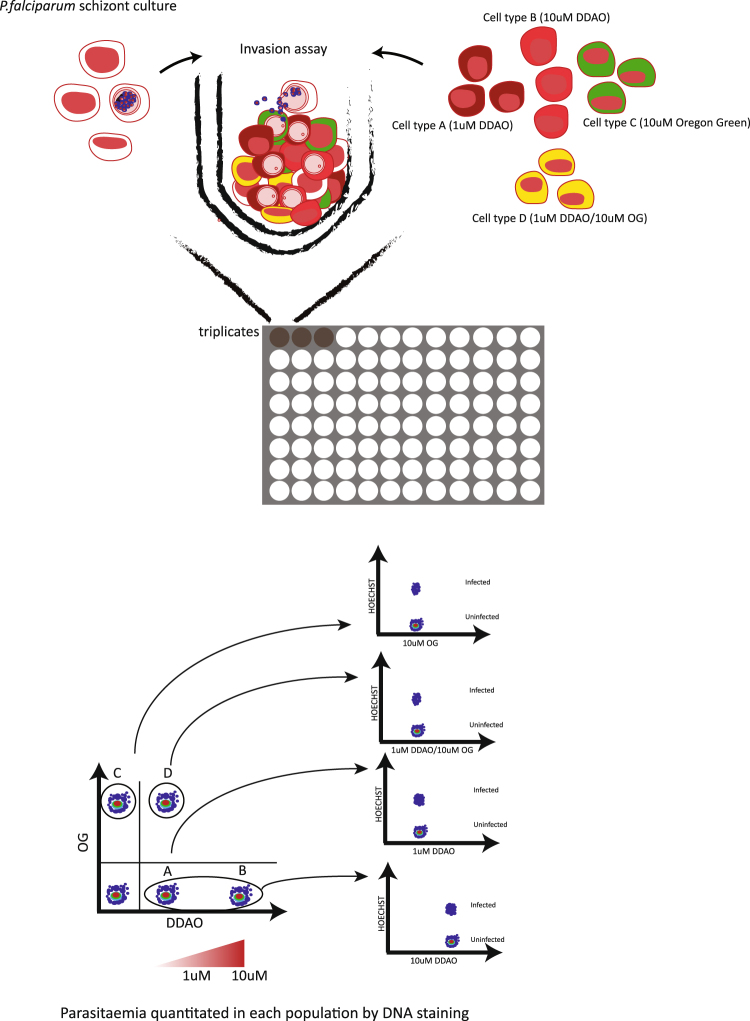
Design of a *Plasmodium falciparum* erythrocyte preference assay.

The utility of fluorescent labelling to mark erythrocytes before they are invaded by *Plasmodium* parasites is now well-established and has been applied to a range of research questions. To extend the methodology we sought to explore how many fluorescent dyes could be combined in a single assay, in order to develop an assay in which labelled erythrocytes from multiple sources could be co-incubated in the same well with *P. falciparum* parasites. Quantifying *P. falciparum* parasitemia in each erythrocyte sub-population would provide a measure of *P. falciparum* erythrocyte preference (Fig. 1), and would present a significant advantage over approaches in which each erythrocyte sample is present in a different well, where technical variation between wells could overwhelm subtle phenotypic differences. An analogous approach has previously been used with two labelled erythrocyte sub-populations to compare invasion into iron deficient and iron replete erythrocytes^17^. To expand the approach to more complex situations such as human blood groups, we tested multiple dyes for their ability to be combined in a single assay.

**Figure 2:**
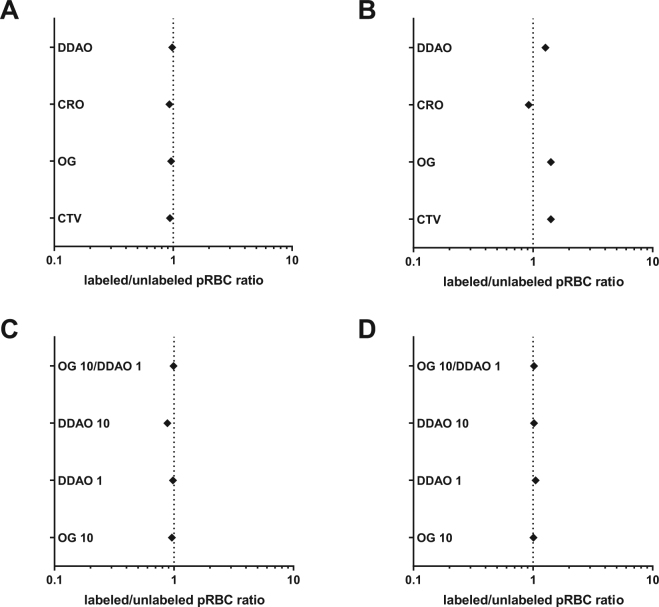
Selection of an optimal cell labelling combination for multiplex invasion of erythrocytes by *P. falciparum*. The impact of labelling erythrocytes with fluorescent dyes on infection by *P. falciparum* strain Dd2 was determined by flow cytometry. (**A**,**B**) Erythrocytes labelled with either CellTrace™ Far Red (DDAO), Calcein Red-Orange AM (CRO), Oregon Green 488 (OG) or Violet (CTV) were incubated individually (**A**) or altogether (**B**). (**C**,**D**) Erythrocytes labelled with 1 or 10 µM of DDAO or 10 µM of OG or a combination of both were incubated individually (**C**) or altogether (**D**). All cells were incubated in the presence of *P. falciparum* parasites for 48 h to allow rupture of schizonts and subsequent invasion of labelled and unlabelled erythrocytes. The cells were then stained with Hoechst 33342 and analyzed by flow cytometry to determine the parasitemia of each population and calculate the labelled invaded erythrocyte/unlabelled invaded erythrocyte ratio. Values greater than 1.0 indicate an increased susceptibility to invasion than unlabelled erythrocyte, while values smaller than 1.0 indicate an increased resistance to invasion.

Erythrocytes were labelled with CellTrace™ Far Red (DDAO), Calcein Red-Orange AM (CRO), Oregon Green 488 (OG) or Violet (CTV) for two hours, then washed repeatedly in *P. falciparum* culture medium. The labelled erythrocytes were then mixed 1:1 with a *P. falciparum* Dd2 parasite culture at 1% parasitemia, and co-cultured for 48 hours, during which time the *P. falciparum* parasites could invade either the fluorescently labelled erythrocytes, or unlabelled erythrocytes that were present in the Dd2 starting culture. After 48 hours, Hoechst 33342 staining was used to label infected erythrocytes, and two colour flow cytometry used to quantitate *P. falciparum* infection into labelled and non-labelled erythrocyte populations. In all cases the ratio of invasion into labelled/unlabelled erythrocytes was indistinguishable from 1, meaning that none of the four dyes had an effect on *P. falciparum* invasion rates (Fig. 2A). To test whether a combinatorial assay was possible, samples of the four labelled erythrocyte populations were mixed together 1:1:1:1:1 with a population of Dd2 parasites which also contained unlabelled erythrocytes. Invasion events occurred in all five erythrocyte populations (Fig. 2B), but in this case the ratios of invasion into labelled/unlabelled populations deviated in some cases from 1.

While all four dyes therefore can be used for *P. falciparum* invasion studies, combining all four in a single well proved challenging, in part because the close proximity of the emission spectra for CTV and OG made developing a precise gating strategy to deconvolute each population difficult (data not shown). We therefore explored whether erythrocytes labelled with different concentrations of the same dye could also be distinguished by flow cytometry. Erythrocytes were labelled with 1 or 10 µM of DDAO, 10 µM of OG, or a combination of both 1 µM of DDAO and 10 µM of OG, and mixed 1:1 with *P. falciparum* Dd2 parasites as described above. Again, there was no difference in invasion rates into labelled erythrocytes relative to unlabelled erythrocytes (Fig. 2C), and in this case the same was also true when all four labelled erythrocytes were co-incubated in the same well (Fig. 2D).

**Figure 3:**
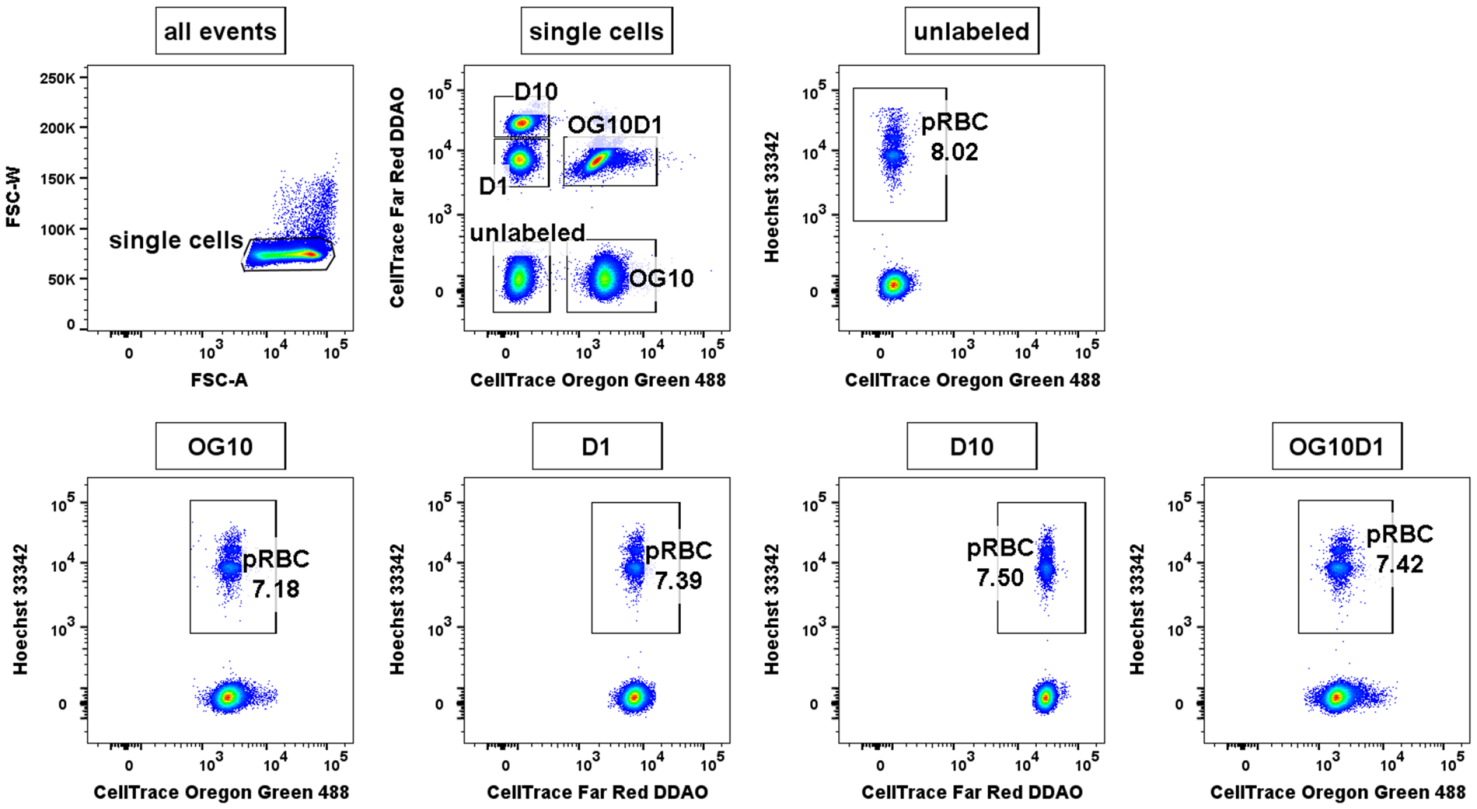
Example gating scheme to determine parasitemia in labelled and unlabelled erythrocytes. Erythrocytes labelled with 1 or 10 µM of DDAO, 10 µM of OG or a combination of both were mixed and incubated with *P. falciparum* parasites for 48 h to allow rupture of schizonts and subsequent invasion of labelled and unlabelled erythrocytes. The cells were then stained with Hoechst 33342 and analyzed by flow cytometry to determine the parasitemia of each population. Individual erythrocyte populations were gated based on their MFI for both OG and DDAO, after excluding debris and doublet events.

To define a final erythrocyte preference assay that gave the most clear separation of emission spectra, and provided the simplest gating strategy, we used the combination of dyes and concentrations outlined in Fig. 2D and shown graphically in Fig. 1. Erythrocytes were labelled with 1 µM of DDAO, 1 µM of DDAO, 10 µM of OG, and a combination of both 1 µM of DDAO and 10 µM of OG, and after co-incubation with *P. falciparum* parasites, infected erythrocytes were labelled with Hoechst 33342. After flow cytometry, single erythrocytes were gated using forward scatter, then unlabelled and the different labelled erythrocyte populations were gated based on a combination of OG and DDAO staining (Fig. 3). The proportion of infected erythrocytes in each labelled and unlabelled population was finally determined using Hoechst 33342 intensity.

### Testing erythrocyte preference across ABO Blood group types

As a test of the erythrocyte preference assay we explored the association between ABO blood groups and erythrocyte invasion. Blood from 40 healthy and unrelated donors, 10 each of blood group A+, B+, O+ and AB+, were obtained from the UK NHS Blood and Transfusion, with appropriate ethical approval. Each blood sample was stained with one dye, and each labelled sample was mixed with blood from the other three blood groups, creating mixtures that contained equal amounts of labelled A+, B+, O+ and AB+ erythrocytes. Samples were mixed in a systematic manner so that each donor blood was used in at least three different combinations, no donor was paired with another donor in more than one combination, and in total 30 unique combinations of A+/B+/O+/AB+ erythrocytes were generated.

**Figure 4:**
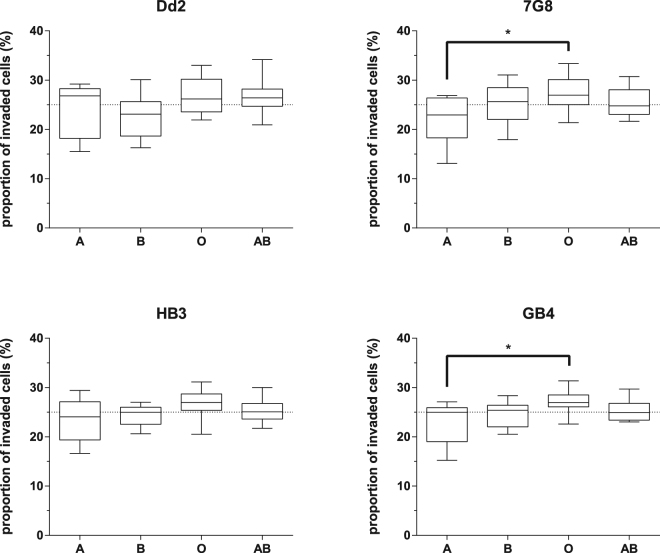
*Plasmodium falciparum* strains show a preference for invading O+ erythrocytes over A+ erythrocytes. The preference of four *P. falciparum* strains for one or more of the ABO blood groups was assessed using an erythrocyte preference assay. Blood group A+, B+, O+ and AB+ erythrocytes were labelled with 1 or 10 µM of DDAO, 10 µM of OG, or a combination of both and mixed so that each well contained erythrocytes from all four blood groups. The erythrocyte mixtures were co-incubated with *P. falciparum* strain Dd2, 7G8, HB3 or GB4 parasites for 48 h to allow rupture of schizonts and subsequent invasion of labelled erythrocytes. Parasitemia was determined by flow cytometry after staining with Hoechst 33342, and the proportion of invaded erythrocytes was calculated as a percentage of total invaded labelled erythrocytes in each well. 40 different healthy volunteers, 10 for each blood group, were used, and each individual blood sample was tested in 3 different combinations of A+, B+, O+ and AB+, with 30 total combinations tested. Statistical significance was determined using one-way ANOVA with Tukey’s multiple comparison test (*p < 0.05).

Each of these 30 unique A+/B+/O+/AB+ erythrocyte combinations was then co-incubated with four different *P. falciparum* strains, broadly representative of *P. falciparum* geographical diversity across Africa, Asia and the Americas – 7G8 was originally isolated from Brazil, GB4 from Ghana, Dd2 from Southeast Asia and HB3 from Honduras. Each combination of erythrocytes was tested in triplicate against all four strains, making a total of 360 assays. After 48 hours, each well was stained with Hoechst 33342, and *P. falciparum* invasion rates into each population quantitated using the gating strategy outlined above (Fig. 3). Parasitemia rates fluctuated between wells and between parasite strains, so to compare across assays, invasion within each well was compared to a theoretical 1:1:1:1 distribution between the four labelled erythrocytes, which is what would be expected if there was no preference between blood groups. After averaging across all 30 combinations, both 7G8 and GB4 strains had a clear preference for O+ erythrocytes over A+ erythrocytes (Fig. 4, p < 0.05). The same trend in preference for O+ erythrocytes over A+ erythrocytes was also seen with the HB3 strain and to a lesser extent with Dd2, suggesting that it may be true across many *P. falciparum* genetic backgrounds, although the trend did not reach statistical significance in these cases (Fig. 4).

**Figure 5:**
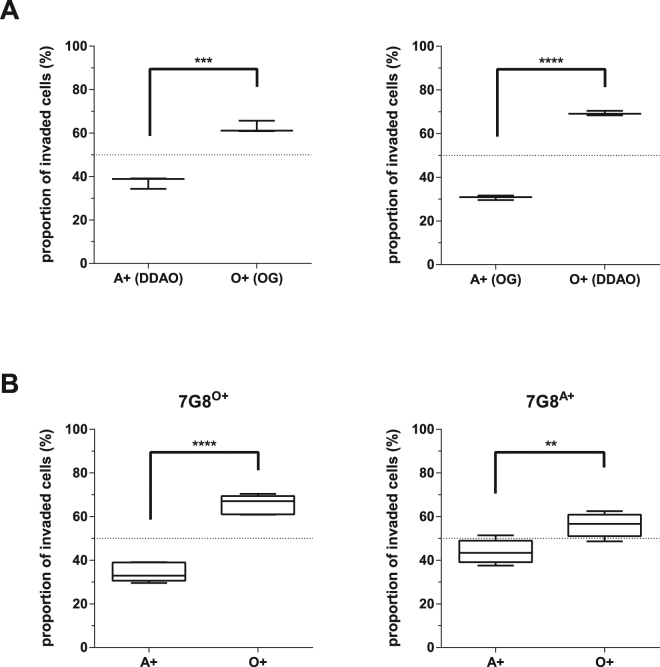
The preference of *Plasmodium falciparum* strain 7G8 invasion for O+ erythrocytes is independent of fluorescent label or previous culture conditions. Erythrocytes from one individual each of blood group A+ and O+, labelled with DDAO or OG respectively, were co-incubated with *P. falciparum* strain 7G8 for 48 h to allow rupture of schizonts and subsequent invasion of labelled erythrocytes. (**A)** The impact of the cell label staining on erythrocyte preference was tested by comparing invasion rates when A+ erythrocytes were labelled with DDAO and O+ erythrocytes labelled with OG, or when the same erythrocyte samples were labelled in the opposite combination, A+ being labelled with OG and O+ with DDAO. (**B**) *P. falciparum* strain 7G8 parasites were cultured in blood group O+ (7G8^O+^) or A+ (7G8^A+^) erythrocytes for >2 weeks before carrying out an erythrocyte preference assay, to determine whether impact of previous culture conditions on erythrocyte preference. In both cases statistical significance was determined using unpaired t test (**p < 0.01, ***p < 0.001, ****p < 0.0001).

As noted above, the assay was set up to limit any potential effect of dye on erythrocyte preference, and our previous experiments had found no evidence of such an effect. Nevertheless, to rule out a specific interaction between a specific ABO blood group and fluorescent dye labelling, erythrocytes from one individual each of blood group O+ and A+ were stained separately with DDAO and OG. 7G8 parasites were then co-incubated with either A+ (DDAO stained) and O+ (OG stained), or A+ (OG stained) and O+ (DDAO stained). In both combinations, the parasites showed a clear and statistically significant preference for O+ erythrocytes over A+ erythrocytes (Fig. 5A).

Another possible confounder could be that the parasites used in the preference assays had previously been maintained in O+ erythrocytes, raising the possibility that they had somehow “adapted” to that blood group, and the results of that adaptation were reflected in the preference assay (although no such adaptation has previously been commented on in the literature, and in some labs *P. falciparum* parasites are routinely swapped between ABO blood groups depending on the availability of blood). To investigate this, 7G8 parasites were cultured in either O+ or A+ erythrocytes for >2 weeks before the erythrocyte preference assay was carried out. In both cases, 7G8 still had a statistically significant preference for O+ over A+ erythrocytes, regardless of which blood group they had been growing in for the last two weeks (Fig. 5B). The extent of the preference for O+ erythrocytes did seem reduced after extended culture in A+ erythrocytes (with an associated reduction in p-value), although variation in the extent of the O+/A+ preference between independent experiments was observed in other instances (for example Fig. 4A) and comparing between wells rather than within wells (as the preference assay is designed to do) always has some risk.

## Discussion

This analysis, of blood from 40 different donors in 30 different combinations against 4 different *P. falciparum* strains, is to our knowledge the largest systematic functional test to date of the effect of any erythrocyte phenotype on *P. falciparum* invasion. The data was generated from only eight 96-well plates of *P. falciparum* culture, emphasising the scalability of the assay. While scaling further is certainly possible, limitations include availability of samples and the complexity of assay design. We endeavoured to eliminate confounding effects wherever possible, such as making sure that although each donor sample was used in three different combinations, it was never matched with another donor more than once. This is clearly the preferable approach to take, but made plate set up difficult and protracted, and therefore potentially prone to error. Scaling to larger numbers of *P. falciparum* strains or erythrocyte samples is therefore possible, but would be aided by automation. We also only tested co-culturing a maximum of four different labelled populations in a single well. Given that four different dyes, and in the case of two dyes, two different concentrations of the same dye, can all be used to label erythrocytes before successful invasion by *P. falciparum* parasites, higher order multiplexing is definitely possible if a rigorous gating strategy can be defined. The assay should therefore be applicable to most human blood group systems.

When the erythrocyte preference assay was applied to a panel of A+/B+/O+/AB+ erythrocytes from 40 independent donors, a clear preference for O+ over A+ erythrocytes was seen in two *P. falciparum* strains, with a trend in the same direction for another two strains. It is important to emphasise that this is not an absolute requirement, but a preference. *P. falciparum* parasites are routinely cultured in O+ erythrocytes in most published reports, but at least in our laboratory we have at times used both A+ and B+ erythrocytes, and informal enquiries of other investigators suggest that the same is true in many labs. The effect is therefore definitely not absolute and is in fact relatively subtle, with invasion into O+ erythrocytes 109% and 108% of expected rates for 7G8 and GB4 respectively, and invasion into A+ erythrocytes 89% and 92% of expected rates. This is exactly the kind of subtle effect that the assay was designed to reveal, and which could potentially be lost in statistical noise if the blood groups had each been present in separate wells, where technical variation in pipetting or other manipulations could easily drown out the signal. The effect was consistent across 30 different combinations of independent donors, and in the case of 7G8, independent of any effect of fluorescent label or prior culture conditions, although there was an indication that the extent of the preference may diminish after prolonged culture in A+ erythrocytes. Whether extending the length of time that the parasites were cultured in A+ erythrocytes would eliminate that preference entirely is not known, but would be interesting to test.

The *in vitro* preference of several *P. falciparum* strains for O+ erythrocytes over A+ erythrocytes is therefore slight but consistent and robust. How does this match with what is known about the interaction between *P. falciparum* and the ABO blood system? The association between ABO and malaria has been investigated extensively in the past, and these studies have consistently shown that Group O is associated with protection against severe malaria^18–20^. This would at first glance would appear to run counter to our findings. It is firstly important to note the general potential for discrepancies between *in vivo* and *in vitro* data. Any attempt to define mechanisms of protection for malaria invariably must involve *in vitro* assays, and there is always the possibility that these simply to do not provide a completely accurate model for *in vivo* malaria. This is unavoidable of course, and almost all of *P. falciparum* molecular and cell biology, as well as many aspects of drug and vaccine development, are built on *P. falciparum in vitro* culture. Given that this culture system, while extraordinarily useful, will always by definition be a model, there is no doubt that for some of the more complex host-parasite interactions it will not alone be an accurate proxy for the reality *in vivo*. It is also important to emphasise that only four *P. falciparum* strains were tested here. While the same preference was seen in two of them, with a clear trend in at least one other, it is inevitably risky to extrapolate to the behaviour of all *P. falciparum* parasites.

Leaving these limitations to one side, what could explain the discrepancy between the known protective effect of group O, against the observed preference of at least two *P. falciparum* strains for this blood type? While all ABO alleles are globally distributed, the frequency of blood type O is generally higher in malaria endemic areas, being at high frequencies in much of sub-Saharan Africa and nearly ubiquitous in South America^18^. In Asia distributions are more intermediate, but there is some evidence that rates of Group O are increased closer to the equator, where malaria transmission rates are higher^18^. The association between Group O and malaria is by no means absolute, but there is an argument that Group O erythrocytes would be the ones that *P. falciparum* would most frequently encounter along the majority of their global distribution, and would definitely have been the most frequently encountered by the progenitors of the GB4 and 7G8 strains in Ghana and Brazil, respectively, which had the strongest preference in our assays. The fact that some *P. falciparum* parasites may have evolved a preference for Group O erythrocytes therefore makes sense from the perspective of parasite fitness - *P. falciparum* parasites have simply adapted to make use of the most abundant ABO blood group present in most malaria endemic regions. That this preference does not lead to increased severity of malaria, and in fact leads to the opposite, suggests that other mechanisms must underlie the protective effect of group O. Indeed, several such invasion-independent mechanisms have been previously proposed, involving reduction in cytoadherence between *P. falciparum* infected erythrocytes and uninfected erythrocytes, platelets or endothelial cells^18,19^, or increased phagocytosis^21^. We therefore believe that rather than being contradictory, these data uncover for the first time the complex co-evolutionary landscape behind the interaction between malaria and the ABO blood group.

As well as shedding new light on the interaction between *P. falciparum* parasites and the ABO blood system, this data presents a robust and potentially widely useful erythrocyte preference assay. As large scale genome wide association studies reveal more variants associated with protection against severe malaria^3^, new assays will be required to investigate protective mechanisms. As the size of the association datasets grow, there will be an increasing likelihood that variants will be found that are enriched in only specific geographic regions, such as the Glycophorin A-Glycophorin B hybrid recently identified to be present essentially only in East Africa^5^. Such findings lead to hypotheses about local interactions between specific human and *P. falciparum* genotypes, which will require testing by combining multiple *P. falciparum* strains and multiple erythrocyte samples. In in order to test hypotheses of this nature, any new assay must be designed with scalability in mind. The erythrocyte preference assay reported here for the first time represents one such scalable approach, and has the potential to be applied to the systematic testing of human blood groups for an impact on *P. falciparum* invasion.

## Methods

### Plasmodium falciparum culture

*P. falciparum* laboratory strains 7G8, Dd2, GB4 and HB3, obtained from Chris Newbold (University of Oxford, Oxford, UK), were routinely cultured in human erythrocytes (NHS Blood and Transplant, Cambridge, UK; informed consent from donors was obtained by NHSBT as part of their recruitment processes) at 5% hematocrit in complete medium containing 0.5% AlbuMAX II (Life Technologies, Paisley, UK), under an atmosphere of 1% O2, 3% CO2 and a balance of N2 (BOC, Guildford, UK). Parasite cultures were maintained synchronized on early stages with 5% D-sorbitol (Sigma-Aldrich, Dorset, UK). Standard Giemsa-stained, blood smear microscopy was performed to determine parasitaemia. Use of erythrocytes from human donors for *P. falciparum* culture was approved by the NHS Cambridgeshire 4 Research Ethics Committee and the Wellcome Trust Sanger Institute Human Materials and Data Management Committee. All experiments were performed in accordance with relevant guidelines and regulations.

### Erythrocyte labelling with intracellular fluorescent dyes

Erythrocytes were labelled with amine-reactive fluorescent dyes. The required volume of erythrocytes at 2% haematocrit in RPMI 1640 (Life Technologies, Paisley, UK) was centrifuged and the pellet resuspended to 2% hematocrit with either 1 or 10 µM CellTrace™ (Thermo Fisher Scientific, Paisley, UK) Far Red (DDAO), 10 µM Oregon Green 488 (OG), 10 µM Calcein Red Orange AM (CRO), 10 µM Violet (CTV), or a combination of 1 µM DDAO and 10 µM OG in RPMI 1640 and incubated for 2 h at 37 °C under rotation. The suspension was washed with complete medium and the pellet resuspended to 2% hematocrit with complete medium and incubated for a further 30 min at 37 °C. The suspension was then washed twice with complete medium and finally resuspended to 2% hematocrit with complete medium.

### Erythrocyte preference assay

Erythrocytes labelled with OG, DDAO, CRO, CTV or a combination of OG and DDAO were added together with parasitized red blood cells (pRBC) at 2% hematocrit to wells of round-bottom 96-well culture plates. Labelled erythrocytes and pRBC were added in equal volumes to make up a culture volume of 100 µL per well at 2% hematocrit and 0.5–1% parasitaemia. The well suspension was then mixed before being incubated for 48 h inside an incubator culture chamber (VWR, Lutterworth, UK), gassed with 1% O2, 3% CO2 and a balance of N2, and kept at 37 °C.

In order to limit the effect of additional confounding variables among donors when incubating pRBC with a mixture of erythrocytes from blood type A+, B+, O+ and AB+, labeled erythrocytes from each donor were incubated with 3 independent sets of erythrocytes from 3 additional donors (one from each other blood type). For example, erythrocytes from donor O1 (blood type O+ donor 1) were mixed with A1, B1 and AB1 in a first well, A2, B3 and AB4 in a second well, and A3, B4 and AB5 in a third well. Erythrocytes from each donor were only mixed once with any of the other donors across all assays. Each co-incubation was carried out in triplicate, and all assays with one strain of parasite were kept to the same 96-well plate. In addition, to ensure that the assay setup had no impact on the preference of individual parasite strains, each assay plate included control wells with erythrocytes used for parasite culture labelled with each of the fluorescent dye and incubated with pRBC individually or concurrently.

At the end of the incubation, individual wells of the erythrocyte preference assay were stained, after washing with RPMI 1640, with 2 µM Hoechst 33342 (Thermo Fisher Scientific, Paisley, UK) in RPMI 1640 for 1 h at 37 °C. The suspension was then washed twice with RPMI 1640, before being fixed with 2% formaldehyde (Thermo Fisher, Loughborough, UK), 0.2% glutaraldehyde (Sigma-Aldrich, Dorset, UK) in PBS for 1 h at 4 °C. Finally, the samples were washed with PBS and resuspended in PBS until data acquisition.

### Flow cytometry and data analysis

Stained samples were examined with a 355 nm 20 mW ultraviolet laser, a 405 nm 50 mW violet laser, a 488 nm 50 mW blue laser, a 561 nm 50 mW yellow-green laser and a 640 nm 40 mW red laser on a BD LSRFortessa flow cytometer (BD Biosciences, Oxford, UK). Hoechst 33342 was excited by the ultraviolet laser and detected by a 450/50 filter. CTV was excited by the violet laser and detected by a 450/50 filter. OG was excited by the blue laser and detected by a 530/30 filter. CRO was excited by the yellow-green laser and detected by a 610/20 filter. DDAO was excited by the red laser and detected by a 660/20 filter. BD FACSDiva (BD Biosciences, Oxford, UK) was used to collect 50,000 events for cell population. FSC and SSC voltages of 423 and 198, respectively, and a threshold of 2,000 on FSC were applied to gate on the erythrocyte population. The data collected was then further analysed with FlowJo (Tree Star, Ashland, Oregon). GraphPad Prism (GraphPad Software, La Jolla, California) was used to plot data generated and perform statistical analysis on the dataset.

The percentage of invasion in each blood group was normalised and calculated as follows. First, % parasitemia was calculated separately for each labelled population in each well, by dividing the number of infected erythrocytes within a given labelled population by the actual number of erythrocytes present in that labelled population, as quantitated by flow cytometry. This controls for any pipetting errors which might result in unequal numbers of labelled erythrocytes within each well. Secondly, the percentage of invasion into each blood group within a given well was normalised and converted to a base of 100 by dividing % parasitemia for each individual blood group by the sum of parasitemias within that well (e.g. if % parasitemia in A^+^ was 5%, B^+^ was 5%, AB^+^ was 4% and O^+^ was 6% in a given well, then %A^+^ was 5/20 or 25%, while %AB^+^ was 4/20 or 20%. This is a critical step that enables comparison across strains. The data for each individual donor blood was then averaged across the technical triplicates for each unique combination of A^+^, B^+^, AB^+^ and O^+^, then across all three combinations of A^+^, B^+^, AB^+^ and O^+^ that contained erythrocytes from this same donor. The data presented is therefore the proportion of invaded cells for each of 10 donors from the same blood group, controlled for the effects of genetic background.
